# New York Letter

**Published:** 1890-10

**Authors:** 


### Correspondence.

### NEW YORK LETTER.

NEW YORK, SEPT, 17, 1890.

Prof. DeGarnes, of this city, at a recent clinical lecture delivered at the New York Post-Graduate Medical School and Hospital, stated that in nine out of ten cases of strangulated vaginal hernia, strangulation existed at the external ring. In his opinion the sac should be opened in all cases except in the most recent and smaller herni, and under proper antiseptic precautions, very little, if any risk, is incurred. Any risk that may be present can be avoided by putting a temporary ligature about the neck of the sac just below the external ring.

If the contents of the sac should be made up of intestine irreducible from adhesions to its side, the utmost care will be necessary in its separation. Masses of protruding omentum should in every instance, be removed, even though reducible, as they are apt to become a potent factor in the return of the hernia. The occurrence of secondary hemorrhage, which he considers one of the most dangerous features in the amputation of omentum, may be avoided by spreading the omentum out and tying each vessel that bleeds, instead of tying the whole stump with a single ligature.

It the treatment of the sac in the case of large hernias, he considers Barker's method the best, and this consists in opening the sac below the external ring, drawing it down as far as possible, and after being sure that it has been freed from its contents, ligating it with silk over the end of the finger which should occupy the neck of the sac until it is closed off from the abdomen. The sac is then divided below the ligature, when retraction of the stump will carry it high up in the canal to the internal ring.

In the case of small and recent hernia, when the sac is thin and appears like normal peritoneum, torsion is recommended as a safe and convenient mode of disposing of it.

In regard to the treatment of the canal, he says, a very great diversity of opinion seems to prevail. Of the McBurney method of procedure, he does not approve, for though he considers the results of this method during the short time it has been in use, very encouraging. The history of scar tissue in other portions of the body leads to the belief that though for a year or two it may be strongest, it is in time certain to be weaker than the surrounding healthy tissue, and lead to a recurrence of the hernia, a result that is apt to leave the patient in a worse condition than before the operation was undertaken. For this reason, he prefers Barker's method of treating the canal, which consists in narrowing its anterior wall by means of heavy black silk sutures applied over its entire length. The wound heals by first intention, leaving the stitches permanently in the tissues. The superficial tissues are at once closed, and the dressings are not renewed for ten days, at the end of which time complete union will be found to have taken place. In the aftertreatment of hernias he does not believe in applying a truss in the case of very small ones, while he considers it perfectly essential in the case of large and medium sized hernial protrusions.

Prof. Seneca D. Powell has for several years been in the habit of using with very satisfactory results, a method of treatment in obscure injuries, attended with pain, but without inflammation about joints, and in muscles, such as arise from strains, and even in rheumatic affections. This method consists in applying lightly and rapidly at several points over the affected area Pacquelin's cautery at a red heat. He recently presented at a clinic a young man suffering from an obscure pain at the shoulder joint. He applied the cautery over the deltoid muscle, and in the very first application a marked improvement set in. After three or four more applications repeated at stated intervals, the patient was discharged cured of all his symptoms. He considers this the very best method which he is acquainted for the cure of the conditions just referred to.

Dr. Wm. H. Porter, of this city, recommends the following as the best and most efficient treatment for diabetes:

| | | |
|---|---|---|
| ℞ | Fel. bovis inspiss, | ℈ ij. |
| | Quinæ sulph., | gr. xxxx. |
| | Ext. nucis vomicæ, | gr. vi. |

Divide into capsules, No. xx, and take one before each meal.

| | | |
|---|---|---|
| ℞ | Ext. hyosyami, fl., | ʒ iij. |
| | Ext. damiani, fl., | ʒ vi. |
| | Potasii bicarb., | ℥ ss. |
| | Mucilaginis, | ℈ ii. |
| | Aquæ, | ℥ iij. |

M. S. A teaspoonful every three hours.

In the treatment of diabetes he says that restricted diet is not so essential, as restriction is the excess of food, to which these patients are markedly addicted. By regulating their diet in this respect, he considers the use of the above remedies the best and most efficient means of treatment he knows.

Kocher's method of reducing subcoracoid dislocations of the humerus is very much in vogue here. It is done in the following manner: The patient is seated, and the surgeon kneels beside him. If it be the right shoulder that is dislocated, the surgeon grasps with his left hand the arm of the patient just above the elbow. The patient's forearm being flexed to a right angle, the surgeon firmly seizes the wrist with his right hand, then adducting the elbow to the body, he rotates the arm out until firm resistance is encountered. He then brings the elbow forward and upward in the saggittal plane across the chest, and finally rotates the arm inward, placing the patient's hand on his opposite shoulder. This method, when properly applied is always successful, and may be employed without the aid of an anæsthetic.

Dr. Wm. T. Bull, Professor of Surgery at the College of Physicians and Surgeons, was the first to introduce it in this country, and under his direction its employment was commenced at the Chambers St. Hospital, and is now the method in use there to the exclusion of all others. During the first six months of its use at the hospital in seventeen out of twenty-one cases, reduction by this method was effected at the first attempt, while in the other four the bone went back at the second, third, fourth and fifth attempt, respectively. In not one case was an anæsthetic used.

Dr. Satterthwaite, of the New York Post-Graduate Medical School and Hospital, recommends the use of the following remedy in cirrhosis of the liver:

℞ Hydrarg. bichlorid. gr. j.

Ammoniæ muriat. ʒ ii.

Syr. aurantii. ℥ j.

Aquæ, ad., ℥ iij.

M. S. Take a dose three times a day.

He also advises the use of a blister over the affected region if pain is present.

